# Motion-in-depth effects on interceptive timing errors in an immersive environment

**DOI:** 10.1038/s41598-021-01397-x

**Published:** 2021-11-09

**Authors:** Joan López-Moliner, Cristina de la Malla

**Affiliations:** Vision and Control of Action (VISCA) Group, Department of Cognition, Development and Psychology of Education, Institut de Neurociències, Universitat de Barcelona, Barcelona, Catalonia Spain

**Keywords:** Motor control, Sensorimotor processing, Sensory processing, Human behaviour

## Abstract

We often need to interact with targets that move along arbitrary trajectories in the 3D scene. In these situations, information of parameters like speed, time-to-contact, or motion direction is required to solve a broad class of timing tasks (e.g., shooting, or interception). There is a large body of literature addressing how we estimate different parameters when objects move both in the fronto-parallel plane and in depth. However, we do not know to which extent the timing of interceptive actions is affected when motion-in-depth (MID) is involved. Unlike previous studies that have looked at the timing of interceptive actions using constant distances and fronto-parallel motion, we here use immersive virtual reality to look at how differences in the above-mentioned variables influence timing errors in a shooting task performed in a 3D environment. Participants had to shoot at targets that moved following different angles of approach with respect to the observer when those reached designated shooting locations. We recorded the shooting time, the temporal and spatial errors and the head’s position and orientation in two conditions that differed in the interval between the shot and the interception of the target’s path. Results show a consistent change in the temporal error across approaching angles: the larger the angle, the earlier the error. Interestingly, we also found different error patterns within a given angle that depended on whether participants tracked the whole target’s trajectory or only its end-point. These differences had larger impact when the target moved in depth and are consistent with underestimating motion-in-depth in the periphery. We conclude that the strategy participants use to track the target’s trajectory interacts with MID and affects timing performance.

## Introduction

In daily-life we often need timing our actions with moving objects that can travel at different speeds and in different directions (e.g., in the fronto-parallel plane or in depth). In order to succeed in tasks that require synchronizing actions with particular times or locations of moving targets, people would certainly benefit from ascertaining parameters like the target’s speed, time-to-contact (hereafter TTC) or motion direction. Knowledge of such parameters can help people better predict future positions of the object and plan timing responses in advance. Moreover, information such as velocity can be used to update targets’ changing positions^1^ influencing performance in timing tasks^2^. An additional factor that may influence performance when targets move around us in three-dimensional (3D) space, creating motion-in-depth (MID), is the changing distance of the object with respect to the observer. These changes affect the retinal speed of the object even when the 3D motion is constant. Yet, to properly interact with moving targets speed estimates should remain approximately constant.

The use of visual fronto-parallel motion, which does not contain a depth velocity component, has predominated in studies aiming at understanding how we time our motor actions. Consequently, targets moving in linear motion^2–8^ or following parabolic flights on the frontal plane^9,10^ have been extensively used. Thus, despite the relevance of MID for daily life interceptive timing (think of a football goalkeeper, or a baseball batter) the effects of different 3D trajectories on interceptive timing, i.e., when a response is required within a particular time window, have been mostly overlooked by previous studies but, see^11,12^ for some exceptions in coincidence timing and motion extrapolation tasks respectively.

Several monocular and binocular cues both retinal and extra-retinal contribute to the perception of speed and direction in MID. Compared to lateral motion, speed discrimination thresholds for MID are usually higher^13,14^, and prone to individual differences^15^. This is probably because there is an overabundance of available depth cues^16^ that makes predictions on timing performance more difficult. When MID is involved, typical monocular cues include optical expansion^17^, shading and texture gradients^18,19^, kinetic depth cues and motion parallax, among others. The main binocular cues for MID include the change of retinal disparity, and inter-ocular velocity differences^20–22^. However, all these monocular and binocular retinal cues need the support of extra-retinal signals^23^ for the estimation of 3D motion, that is MID. In addition to this diversity of cues, the perceived speed of MID depends on which part of the retina is stimulated^16,24^ and the perceived spatial 3D trajectories or motion extent are affected by well known biases^14,16,25–28^. The variability in the perception of MID, including speed and direction, makes it worth studying the performance of response timing when dealing with objects moving in depth. While most of the studies interested in MID have used perceptual judgements of speed or direction, timing performance has not been tested and it is difficult to predict due to the large number of MID cues and individual differences in processing them. An exception of interceptive timing tasks with motion other than linear or fronto-parallel are all the studies involving parabolic motion in depth (e.g., head-on approach trajectories), which have been motivated by testing specific models usually involving internalized variables like Gravity or physical size^29–35^ rather than the effects of MID themselves.

**Figure 1:**
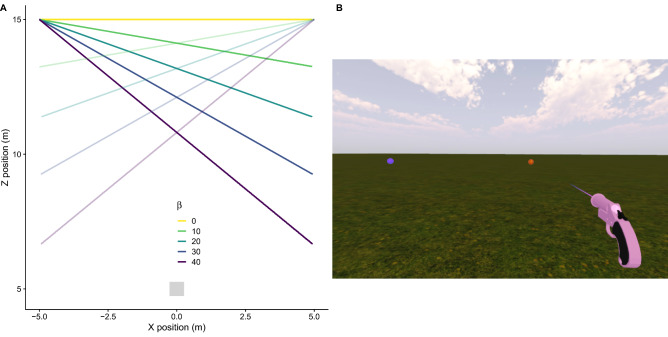
Trajectories in depth and illustration of the task. (**A**) Different possible trajectories described by the moving target. The starting position (x, z) of the target could be (− 5, 15) and then moved rightwards or (5, 15) and moved leftwards (trajectories with alpha transparency). The angle relative to the initial position of the participant (0, 5) could vary from 0° (yellow) to 40° (violet). (**B**) The moving target (red ball) approaches the interception or shooting location (violet) sphere; both target and shooting location have the same simulated physical size. The same grass texture was used in the two conditions (see “Methods”).

In this study we used immersive virtual reality (VR) to look at whether and if so, how, performance (defined as temporal errors) in a shooting task differs across multiple 3D movement trajectories in a shooting task. Figure 1A shows a top-view of the different trajectories used in our Experiment. Trajectories differed in their angle of approach (*β*) that could be 0, 10, 20, 30 or 40 deg. A *β* = 0 deg (yellow trajectory in Fig. 1A) corresponded to fronto-parallel motion. The different spatial paths were combined with three different speeds within the immersive Virtual Reality environment (see Fig. 1B for an illustration of the 3D scenario). Participants had to shoot at a moving target (red ball in Fig. 1B) when it was at an indicated shooting location (violet sphere in Fig. 1B). Since we projected stereoscopic images, both monocular and binocular cues were available and we did not limit the number of these cues or introduce any conflict between them besides the conflicting use of accommodation^36^. It is known that the use of binocular cues (e.g., binocular disparity or change of disparity over time) to recover MID or the different approaching distances often depends on the level of experience of the participant with VR settings^37^ and that non-experienced users would rely more on monocular cues like optical expansion (see the projected size difference in Fig. 1B) that is known to be used when estimating TTC^38^. We did not constraint the tracking strategy (which was inferred from the measurements of head movements only) used by participants in any way, and so was it the one resulting from their own natural behavior or choices in exploring ways of solving the task. By using different tracking strategies people can exploit more foveal or peripheral information. This is important because some relevant cues for MID are perceived differently in the fovea than in the periphery. For example, speed of MID is underestimated in the periphery^24^, suggesting a contribution of inter-ocular velocity differences in speed perception in depth^39,40^.

## Methods

### Participants

Fourteen subjects (age range 22–33, 7 males) participated in the experiment. Twelve of them were right-handed and two were left-handed as by self-report. All of them had normal or corrected to normal vision (with contact lenses), and none had evident motor abnormalities. The study was approved by the ethical committee of the University of Barcelona (IRB00003099) and followed the requirements of the Helsinki convention. All subjects gave written informed consent to take part on the study.

### Apparatus

The experiment was run by an Intel i7-based PC (Intel, Santa Clara, CA). The stimuli were rendered by an NVIDIA GeForce GTX 1070 and sent for display to an HTC Vive Pro head-mounted display (HMD) at 90 Hz per eye (2880 × 1600). The field of view (FOV) of the HMD is 100° (horizontal) by 110° (vertical) for each eye. The head position in 3D coordinates (x, y and z) and the angular information (yaw, pitch and roll) were recorded by two SteamVR BASE STATION 2.0 at 90 Hz. Participant’s responses were registered through one of the headset controllers.

### Stimuli

The virtual scene and stimuli were created with Unity software (Unity technologies, San Francisco, CA). Moving stimuli or targets consisted of a red sphere of 22 cm of diameter (same size as a soccer ball but without familiarity cues) that appeared at one of two possible initial positions (see Fig. 1A). After a period of 2.22 s the target started to move at a constant speed along a straight trajectory. The speed at which the target moved could be either 5.56, 7.14 or 10 m/s and was randomized across trials. The use of different speeds will discourage using positional or distance information alone. The direction of motion (leftwards or rightwards) was also varied on a trial-to-trial basis. Figure 1A shows all the possible trajectory angles (*β*) between the target and the initial position of the observer (grey square at position (0, 5) in Fig. 1A). The angle (*β* in Fig. 1A) could be 0 (fronto-parallel motion), 10, 20, 30 or 40°.

The target moved at the designated speed towards a shooting location denoted by a violet sphere (same size of the target) that was located at a distance of $10/cos\left(\beta \right)$ m, from the initial position of the target (i.e., at 10 m when $\beta =0$ and at 13.05 m when $\beta =40$, see Fig. 1B). The target intersected the shooting location without stopping and continued to move until 1 s after the intersection. The time that it took for the target to reach the shooting location ranged between 1 and 2.34 s across all *β* angles and targets’ speeds.

### Procedure

Each trial started when the participant entered a white square (50 × 50 cm) rendered on the floor at position (0, 5) (shown in grey color in Fig. 1A). Once the sensors detected that the HMD was within this area, a beep indicated that the trial was about to start. The square then disappeared, and both the target and designated shooting location appeared at the same time. After 2.22 s the target started to move towards the designated shooting location and the task for the participant was to shoot at the target and try to hit it when the target was within the shooting location (violet sphere).

If the target was hit while overlapping with the shooting location, the violet sphere turned into a green circle. If the shot reached the designated shooting location (violet sphere) when the target was not there, it turned into a red circle. Pilot sessions showed that the task was quite difficult (less than 20% hits) in terms of getting positive feedback based on the overlapping of target and shooting location. We thus relaxed the temporal constraints to increase the proportion of trials in which positive feedback was provided in order to keep participants motivated. Therefore, we used twice the size of the target as a boundary to define the overlap with the shooting location and give feedback. Participants never reported being aware of this manipulation after questioning them at the end of the whole experiment. Furthermore, participants found the task very engaging and motivating.

### Conditions

The study had two blocked conditions (named *delayed feedback* and *immediate feedback*) that were run in counterbalanced order across participants. The difference between these two conditions consists in the temporal delay to obtain the feedback about the success after the motor response.

#### Delayed feedback

In this condition we examined performance in the shooting task when the feedback of the action was delayed. The shooting task was implemented by triggering a bullet with a gun that was rendered at the same position of the hand-held controller. The bullet was represented by a pink sphere (diameter of 4.4 cm) that travelled at 55 m/s. This speed will introduce a delay between the moment at which the participant pulls the trigger and the moment at which the bullet reaches the target’s path when it is within the shooting location (as would happen when shooting with a real gun). The delay in reaching the designated shooting location when the trajectory had an angle *β*=0° was 0.111 s longer than when the *β* = 40°. Participants were not explicitly told about the delays.

#### Immediate feedback

In this other condition, we replaced the bullet by a simulated instant laser transforming the task in a typical coincidence timing task^2,41,42^. In that case, when participants pulled the trigger they briefly saw a pink line extending from the gun onwards. By using a laser, the differential delayed impact between the different trajectory angles disappeared. That means that the laser immediately intersected the target’s path.

In both conditions targets moved on a fine-grained grass textured background that did not provide any landmark or familiar size cue (see Fig. 1B). Each condition consisted of four sessions of 180 trials each (5 angles × 3 speeds × 2 directions × 6 repetitions) per session. That makes a total of 1440 trials (180 trials × 4 sessions × 2 conditions) per condition. The two conditions were blocked but the order of the type of condition was random across participants. Participants had a break between blocks for as long as they wanted. If they needed to rest during the session, they could also do so by not standing in the square that was projected on the floor at the beginning of each trial. In all cases, participants were told that they were free to move during the trial. Before starting the first session they were shown the spatial boundaries (space dimensions) of the 3D environment, and were aware that a grid appeared on the visual field if they were about to trespass these boundaries.

The motivation underlying the use of these conditions is twofold. The first reason is methodological. The use of the *immediate feedback* condition would allow to see whether any systematic error in the *delayed feedback* condition is only due to the differential time that it took for the bullet to intersect the different trajectories (*β* angles) that the target could move along. The second reason is theoretical. It has been reported that timing a response with a single press (that is, without any time between the response and the feedback) favours the use of spatio-temporal variables (e.g., variables based on first temporal derivatives of spatial information: position or visual angle)^43^ over positional information. In our case, this could result in using the target speed differently when updating the position of the target. This reason also motivates using different target speeds in addition to the fact that using a single speed usually makes participants use positional or traveled distance information.

### Data analysis and hypothesis testing

We registered the time at which participants pulled the trigger in both conditions and the temporal (and spatial) error in each trial. In the *immediate feedback* condition, the time at which the trigger is pulled is the same as the time at which the laser crosses the target’s path. Spatial errors were defined as the difference in position between the laser (or bullet, in the *delayed feedback* condition), at the moment the laser (or bullet) crossed the target’s path. Temporal errors were defined as the time between when the laser (or bullet) crossed the target’s path and when the target’s reached that position. In addition to these variables, we also recorded the orientation and position of the head. Since we did not record eye movements we have to infer, and consequently be cautious, where participants might be looking by checking the head orientation in combination with the field of view of the HMD.

Since we instructed participants to hit the target at the shooting location defined by the violet sphere, we only had temporal errors associated to responses^44^, except for the trials in which subjects missed the shooting location (3.9% of the trials). The temporal error between when the bullet reached the designated shooting location and when the target did so. The trials in which the designated shooting location was missed (3.9%) were excluded from the analysis. The temporal errors and the proportion of hits are the dependent variables we use to define performance.

#### Tracking strategy categorization

We also analyzed the head position and orientation during the target’s motion and categorized every trial into one of two possible groups depending on the head movement. A preliminary visual inspection of head orientation (i.e., the yaw angle) during the target movement in every trial revealed that two possible strategies were used: participants tracked with their heads either the *trajectory* or the *end-point*. In order to automatically detect these strategies we used a threshold in the change of the yaw angle (rotation of the head around the y-axis) to categorize the trials into one of these two strategies. A trial was considered as *tracking the trajectory* if the change in the yaw angle was larger than 20° during the target motion, that is if the head rotated more than 20° during the target motion and the rotation was consistent with the head tracking the target motion. Otherwise, it was considered as *tracking the end-point*. The 20° threshold value was chosen after manually checking a sample of 100 trials across different participants and obtaining a 99% accuracy in classifying the trials as following one or the other tracking strategy.

#### Comparison between conditions

Generally, we performed ANOVAs to test whether approaching angle, target speed and feedback condition (*delayed* vs *immediate*) have any significant effect on the temporal error. ${\chi }^{2}$ based test were used to compare fractions of hits between different conditions. We used Linear Mixed Models (LMM) to estimate parameters (e.g., slopes of temporal errors against angle *β*). ANOVAs were conducted on the LMM outputs when the analyses involved the tracking strategy, since this is a post-hoc variable depending on individual strategies and would likely lead to an unbalanced design, which is better handled by LMM.

From previous literature in TTC and interceptive timing, we know that people tend to commit later temporal errors for faster speeds for both lateral motion^2^ and MID^11,42^. We expected then overall differences in performance depending on the target speed, e.g. later responses for faster targets. Also we looked at whether the differences in speed lead to equivalent differences in the temporal error for the different angles (e.g., different degrees of MID).

Based on previous work, the effect of tracking the target (or, alternatively, looking somewhere else) on timing errors is more controversial. It is known that the speed of targets is better estimated when one directs the gaze at them than when fixating^45,46^ and that eye movements (e.g., smooth pursuit) can help refine an internal representation of the target motion^47^; but this has been shown mainly in 2D or lateral motion. However, tracking the moving object does not necessarily lead to more accurate timing responses or better interceptive performance. For example, using real ball 3D catching^48^ different eye movement strategies led to similar performance. Similarly, studies have shown that errors in an manual interception task (2D motion) did not depend on the quality of pursuit^49^. In these studies, motion was predictable. One key factor for tracking the target to improve task performance seems to depend on the predictability of the target^50^. Pursuit would help estimate speed in cases where motion needs to be extrapolated after an occlusion before the moment of interest^51,52^. Since motion is predictable in our experiment we do not have strong predictions concerning different possible tracking strategies. However, target speed estimates could benefit from tracking the trajectory but the effect on the interception error, like more anticipatory responses, would be mediated by how much weight is given to speed when updating the position of the target^2^.

## Results

### How do participants solve the task?

**Figure 2:**
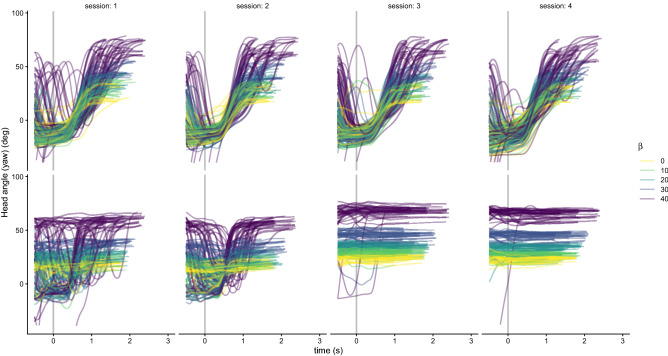
Examples of two participants (#1 and #5 in Fig. 3; bottom and lower panels, respectively) as a function of time. The lines show the Head angle (yaw) for the individual trials of each session (different columns) of the delayed feedback condition. The yaw values have been normalized for the two directions. A yaw angle of 0° corresponds to the head oriented straight-ahead [e.g. looking at (0, 15) from (0, 5)]. The vertical line denotes the stimulus motion onset. The participant in the upper panels always tracks the target’s trajectory with the head, while the participant in the bottom panels initially interleaves two tracking strategies in sessions 1 and 2, and finally fixates on the end-point of the trajectory (end-point tracking strategy). The different colors code the approaching angle *β*.

**Figure 3:**
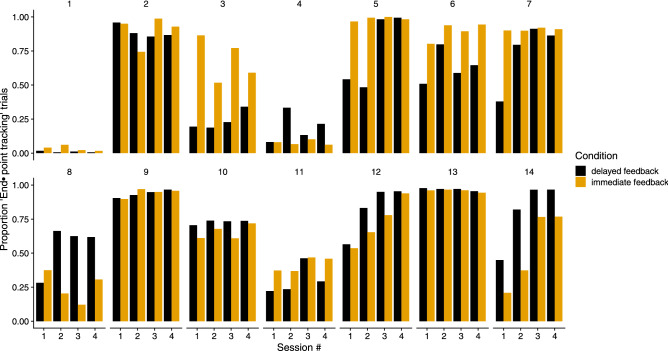
Proportion of trials in which participants adopted the end-point tracking strategy (i.e., the head was oriented towards the shooting location during all the trajectory). This proportion is shown for the two conditions (color coded) and for each participant (different panels) as a function of the session number.

Figure 2 shows the head orientation across time of two participants for all trials and sessions in the *delayed feedback* condition. This figure illustrates the two tracking strategies followed by the participants. The participant in the upper panels (participant #1 in Fig. 3) consistently tracks the target’s trajectory with the head, while the participant in the bottom panels (participant #5 in Fig. 3) tracks both the trajectory and the end-point in the first two sessions and ends up with tracking the end-point in the two last sessions (constant yaw angle through all the target motion).

Figure 3 shows the proportion of times that each participant (in different panels) used the end-point tracking strategy across sessions for the two conditions (color-coded). Participant #1, for example, almost always tracks the trajectory (shown in the upper panels of Fig. 2). This tracking strategy is favoured by participants #1, #4 and #11, as well as by #3 in the *delayed feedback* condition (black bars) and by #8 in the *immediate feedback* condition (yellow bars). Other participants (e.g., #5 in the *delayed feedback* condition, or #12 and #14 in both conditions) predominantly used the end-point tracking strategy. In order to give an idea of the number of trials for the proportions shown in Fig. 3, participant #1, for example, tracked the end-point in 7 trials and the trajectory in 670 trials in the *delayed feedback* condition, while the frequencies in the *immediate feedback* condition were 25 and 685 trials for end-point and trajectory tracking respectively. The number of total trials included in each condition is similar for all participants.

### Effects of angle, speed and feedback condition on the temporal error

**Figure 4:**
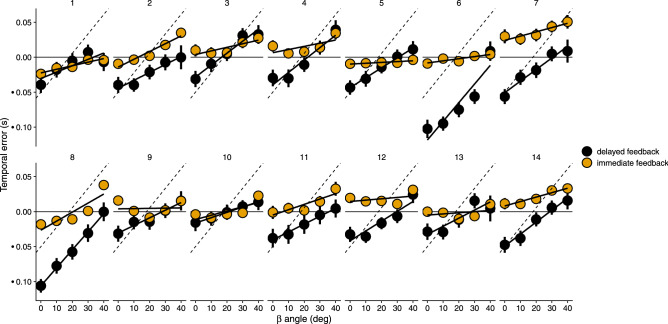
Temporal errors as a function of the *β* angle for the two conditions (color coded). Each participant is plotted on a different panel. The dashed line indicates the errors participants would make if the differential time it took for the bullet to intersect the target’s path in the delayed feedback condition had been ignored. Negative values denote late responses.

Figure 4 shows the temporal error for the two conditions as a function of the angle of approach *β*. Each participant is shown on a different panel. There is a very clear and consistent trend in the temporal error: the error gets more positive (earlier responses) with larger values of the angle *β*. This trend is much clearer in the *delayed feedback* condition (i.e., the simulated bullet). The dashed lines denote what would be the predicted slope of the temporal error if participants had not taken into account the temporal differences of the bullet intersecting the target’s paths for the different angles. Except for participants #6 and #8, the estimated slope of the temporal error is smaller than that predicted if the differential delay had not been considered. However, the *immediate feedback* condition also shows a significant trend of the temporal error despite the laser intersecting the target’s path at the same time as the trigger is pulled (and thus, with no delay for the different trajectory angles).

**Figure 5:**
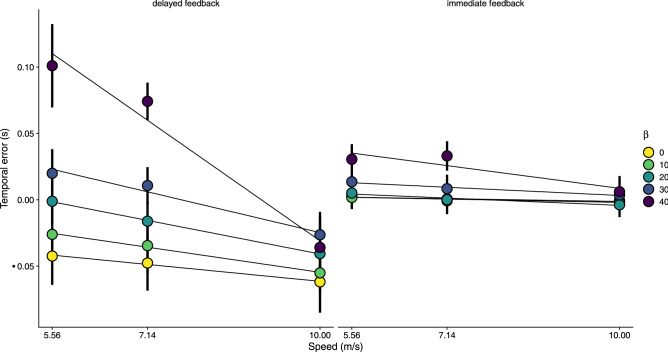
Temporal error as a function of target speed for the two conditions (different panels) and split per approaching angle of trajectory (*β*, color-coded). Negative values denote later responses.

We used three different target speeds and wanted to know whether they affected the temporal error. Figure 5 shows the average temporal error against target speed for the different *β* angles and the two feedback conditions. By visual inspection one can directly see clear differences in the pattern of temporal errors between the two feedback conditions. As expected, faster speeds provoke later temporal errors and the effect of speed on the temporal error is larger in MID (notice how the error changes as a function of speed for the different values of the *β* angle).

In order to test the significance of the effects of all these independent variables, we ran an full ANOVA on the temporal error with speed, feedback condition and *β* as within subjects variables. Both feedback condition (${F}_{\left(1,13\right)}$ = 23.24, p < 0.001) and the *β* angle (${F}_{\left(1,13\right)}$= 93.05, p < 0.0001) had a significant effect on the temporal error, but the speed did not have a significant main effect (${F}_{\left(2,26\right)}$ = 0.34, p = 0.71) on the temporal error. Yet, the interaction between speed and angle of approach (*β*) was significant (${F}_{\left(2,26\right)}$ = 31.7, p < 0.001) as well as the triple interaction speed × angle × condition (${F}_{\left(2,26\right)}$ = 13.28, p < 0.001). These results reflect a larger effect of speed (e.g., later responses for faster speeds) as the *β* angle gets larger (double interaction) and that this trend is much stronger in the *delayed feedback* condition (triple interaction). The larger effect of target speed on the temporal error in the *delayed feedback* condition suggests that speed can be used differently in the two conditions. Since the final temporal error is basically determined at the moment of the response, it will be worth looking at the position of the target when the response was triggered (see positional analysis below).

The interaction between feedback condition and angle was also significant (${F}_{\left(1,13\right)}$ = 57.22, p < 0.001). This is consistent with the slope of the temporal error as a function of *β* in the *delayed feedback* condition being larger than the slope in the *immediate feedback* condition. Importantly, the estimated slope of *β* (0.51 ms/°) in the *immediate feedback* was significantly larger than zero (t(13) = 5.42, p < 0.001), which denotes a significant contribution of the *β* angle to the temporal error in this condition. To further test whether this effect is significant we compared two nested linear models in the *immediate feedback* condition: a null model with only the intercept and a model with *β*. The model that includes *β* was better than the null model (likelihood ratio test: ${\chi }^{2}\left(1\right)$ = 36.563, p < 0.001).

### Effect of the tracking strategy on performance

The effect of the angle trajectory on the observed bias in the temporal error is clear and very consistent across participants. The larger effect in the *delayed feedback* condition could suggest that participants did not fully account for the differential time delay of the bullet in the different angles. It seems that some of the participants did not compensate at all since the slope appears to be very close to that predicted by the differential travel delay (see Fig. 4). However, the results in the *immediate feedback* condition (in which there is no differential travel delay between angles) are also clear and suggest that the temporal bias is caused by the angle of approach rather than by whether the differential delays across trajectory angles are considered.

**Figure 6:**
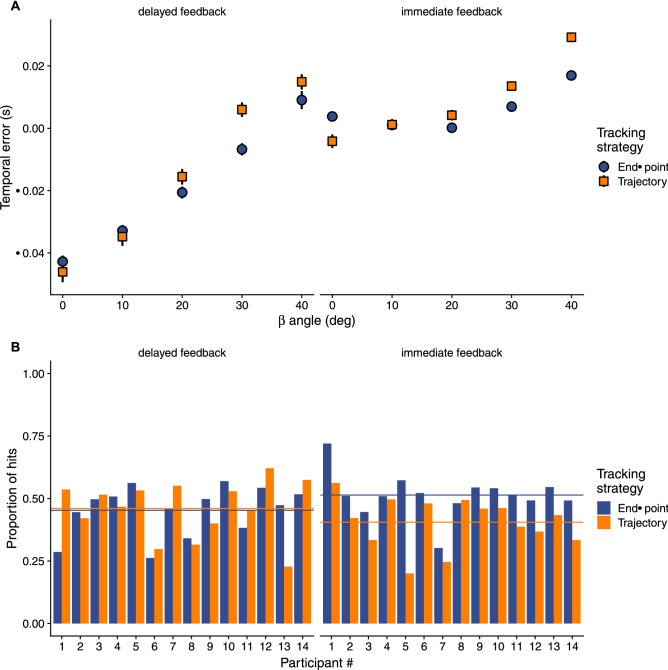
(**A**) Mean temporal error as a function of the angle of approach for the two conditions (in different panels) and split per tracking strategy (color and shape coded). Error bars denote 95%-CI. (**B**) Proportion of hits (a hit was scored whenever the target was intercepted while overlapping with the sphere representing the interception zone) per participant in the two feedback conditions (different panels) and split by the tracking strategy (color coded) in individual trials. The horizontal lines denote the mean proportions of hits across participants.

Figure 6A shows the mean temporal error per angle of approach and the two tracking strategies. Although there is a trend for earlier (more positive) responses when people tracked the trajectory, the strategy did not have a significant effect on the temporal error (${F}_{\left(1,247\right)}$ = 0.63, p = 0.43). The interaction *β* × tracking strategy failed to reach significance too (${F}_{\left(1,247\right)}$ = 2.61, p = 0.11).

In terms of hits there was no significant difference between the two conditions (Fig. 6B). The proportion of hits was 47% in the *delayed feedback* conditions versus 48% in the *immediate feedback* condition (${\chi }^{2}\left(1\right)$ = 1.6, p = 0.2). We also looked at the effect of the tracking strategy within each condition. The tracking strategy did not make any difference in the *delayed feedback* condition (47.3% of hits when adopting the end-point strategy versus 47.7% when tracking the trajectory, ${\chi }^{2}\left(1\right)$ = 0.1, p = 0.76). However, in the immediate feedback condition the end-point tracking strategy led to a larger proportion of hits than the trajectory tracking strategy (50.1% versus 45.3%, ${\chi }^{2}\left(1\right)$ = 19.4, p < 0.001). This could be the result of the earlier responses when tracking the trajectory. Since the tracking strategy is a post-hoc variable that depends on individual participants and, therefore, is not balanced (see Fig. 3) we conducted the same proportion comparison analysis but based on randomly sampling (3000 times) the same number of trials (2500) per strategy and fitted the ${\chi }^{2}$ density function to obtain the mean value of the ${\chi }^{2}$ statistic. Again, the difference between tracking strategies was not significant in the *delayed feedback* (${\chi }^{2}\left(1\right)$ = 0.48, p = 0.49), but the significant difference held in the *immediate feedback* condition (${\chi }^{2}\left(1\right)$ = 11.51, p < 0.001).

#### Positional analysis

**Figure 7:**
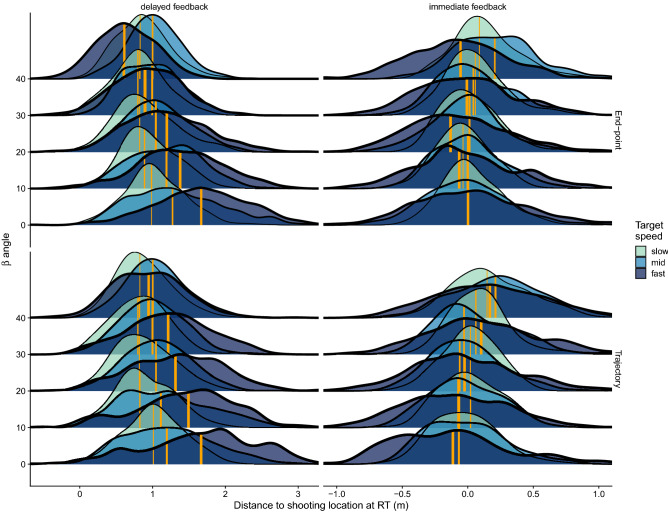
Distributions of distance between target and the shooting location at the moment of the response for the two conditions (column-wise panels) and tracking strategy (row-wise panels). Within each panel we show the densities for the different angles (*β* values) and target speeds (different fill color and line width). The vertical orange lines denote the medians of the corresponding distribution (the thicker the line the faster the speed).

Figure 7 shows the distributions of the distance between the target and the shooting location at the time the trigger was pulled (reaction time, RT). The distribution is shown separately for each angle, condition and speed. Since we are including the tracking strategy as a variable in addition to speed and *β* angle, we conducted an ANOVA on the distance to the shooting location for each condition separately in order to make the interpretation simpler. The ANOVA was run on a LMM (see Methods). The model included the *β* angle, the target speed and the tracking strategy as fixed effects, and participants were considered as random effect.

In the *delayed feedback* condition, the ANOVA yielded very significant main effects of approaching angle (*β*) (${F}_{\left(1,366\right)}$ = 124.4, p < 0.001), target speed (${F}_{\left(1,366\right)}$ = 77.4, p < 0.001) and the interactions $\beta \times$ tracking strategy (${F}_{\left(1,366\right)}$ = 9.2, p = 0.0026) and $\beta \times$ speed (${F}_{\left(2,366\right)}$ = 36.2, p < 0.001). No other effect was significant. In the *immediate feedback* condition similar significant effects were obtained except for the effect of target speed which was marginal (${F}_{\left(2,357\right)}$ = 2.85, p = 0.06). The angle of approach was significant (${F}_{\left(1,358\right)}$ = 38, p < 0.001) as well as the interactions $\beta \times$ tracking strategy (${F}_{\left(1,357\right)}$ = 10.12, p = 0.0015) and $\beta \times$ speed (${F}_{\left(2,357\right)}$ = 6.1, p = 0.0025). The effect of the tracking strategy then was not significant in either condition but it interacted significantly with *β* in both of them. Next, we will elaborate on these different effects.

In the *delayed feedback* condition, people pulled the trigger when the distance between the target and the shooting location was larger for faster speeds. This makes sense because faster targets will travel longer distances than slower ones for a similar arrival time to the shooting location. Yet, this anticipation did not fully compensate for speed differences and made participants be 23 ms later in the faster speed than in the slower one (t(1) = 8.59, p < 0.001). However, the positional difference between speeds becomes smaller as the MID increases (i.e., for larger *β*) as can be noted by the separation between the medians of the distributions for the different speeds (orange vertical lines). In addition, when targets moved in depth (larger angles) people responded (i.e., pulled the trigger) when the distance between the target and the shooting location was smaller. This is consistent with compensating for the travel delay of the bullet. Interestingly, however, the rate at which the distance was shortened with *β* in the *delayed feedback* condition, was different depending on the tracking strategy. When tracking the end-point participants responded, as *β* increased, when the target got progressively closer at a rate of 1.18 cm per deg of *β* (0.56, 0.7 and 2.29 cm/° for the slow, mid and fast speed respectively). This rate (i.e., the slope of the distance to the shooting location against *β*) was smaller (0.3 cm/°) when participants tracked the trajectory (0.1, 0.6 and 0.2 cm/° for the slow, mid and fast speed, respectively) consistent with the significant interaction *β* × tracking strategy reported above. This different pattern between the two strategies can be explained by participants perceiving MID differently in the fovea than in the periphery. More concretely, the shorter distance of the target to the shooting location when participants tracked the end-point and MID was present is consistent with underestimating the speed in the periphery^24^.

A slightly different pattern is observed in the *immediate feedback* condition. Figure 7 shows how the distance between the target and the shooting location is more similar across speeds (the main effect of speed was marginal). Since the response time is the same as the interception time in this condition, this similarity is, in part, trivial. However, the rate of change of the distance with the *β* angle and the effect of the tracking strategy is also different. Unlike in the *delayed feedback* condition, the average distance of the target to the shooting location increased with *β* rather than decreasing. Note the progressive rightward shift of the vertical lines (i.e., of the medians of the distributions) in the *immediate feedback* condition. When tracking the end-point, the distance to the shooting location increased at an average rate of 0.17 cm/° (0.2, 0.4, − 0.09 cm/° for the slow, mid and fast speed respectively). Like in the *delayed feedback* condition, the distance increased with *β* at a larger rate (0.28 cm/°) when participants tracked the trajectory consistently with the significant interaction between *β* and tracking strategy reported above for the *immediate feedback* condition. This is again consistent with participants perceiving slower the speed in depth in the periphery, or faster when they tracked the trajectory (more foveal vision). The difference, however, is smaller (end-point strategy: 0.17 cm/° vs trajectory strategy: 0.28 cm/°) than in the *delayed feedback* condition. The fact that we obtain smaller changes of distance between the target and the shooting location with *β* in the *immediate feedback* condition can be explained by the fact that the approaching angle *β* does not introduce any travel delay. However, the differences between tracking strategies cannot be explained by these delays, but are consistent with previously reported MID effects on perceived speed.

#### Position variability

In addition to the mean distances between the target and the shooting location at the moment at which the trigger is pulled, Fig. 7 also reveals a different pattern regarding positional variability between the two feedback conditions. One can notice how in the *delayed feedback* condition the width of the distributions seems to narrow as *β* increases. This effect of the angle of approach is not clearly noticeable in the *immediate feedback* condition; the width of the distributions does not seem to change with *β*. After computing the SD of the individual distributions across feedback condition, angle of approach and target speed, we conducted an ANOVA on the SD of distance distribution with these variables as independent variables. The main effect of *β* was significant in the *delayed feedback* condition (${F}_{\left(1,13\right)}$ = 41.45, p < 0.001) but not in the *immediate feedback* condition (${F}_{\left(1,13\right)}$ = 0.002, p = 0.967). The average distance variability did not change with the *β* angle when the feedback was immediate (slope not significantly different from zero: slope = $-8.836\times {10}^{-06}$, t(1) = − 0.020, p = 0.984), but it did significantly in the *delayed feedback* condition (slope = − 0.243 cm/°, t = − 3.944, p < 0.001). The positional SD then decreased at a rate of 0.243 cm/°. The target speed had a very significant effect in both conditions (${F}_{\left(1,13\right)}$=97.61, p < 0.001 and ${F}_{\left(1,13\right)}$=120.3, p < 0.001 for the *delayed* and *immediate feedback* conditions respectively). This is expected and well known in the interception literature as increasing speed makes positional judgements more difficult^53^.

## Discussion

In the present study we have shown that MID has an effect on the temporal errors people make in an interceptive timing task (shooting task in our case) that is very consistent across participants and conditions. When compared with fronto-parallel motion, performance when MID is present shows earlier final temporal errors. This conclusion is based on the fact that increasing the angle of approach (*β*) leads to more anticipatory responses. This pattern is present in the two studied feedback conditions: *delayed* and *immediate feedback*. Although part of this trend can be accounted for by the travel delay of the simulated bullet (i.e., the bullet reaches the target’s path earlier as the angle *β* increases), in the *immediate feedback* condition there is no such delay and the trend is very significant and consistent across participants too. Apparently, participants failed to fully recover the 3D speed when MID was present, in a similar way as failure of velocity constancy has been shown^54^ when there is a lack of familiar-size cues that could have otherwise helped scale the changing distance across the different angles, specially in VR settings^55^. This effect in an interceptive timing task is consistent with more anticipatory errors for trajectories approaching the observer (MID) than for fronto-parallel ones in a motion prediction task reported previously^12^.

Accurate perception of MID (both speed and direction) is a complex process that requires integrating extra-retinal (e.g., gaze direction) and retinal information so that 2D information from the retinae are properly mapped to a 3D layout, but this transformation can be incomplete^16^. It is clear that the differences in temporal errors as a function of speed depended on the angle of approach, as shown in Fig. 5. The effect of the *β* angle, regardless of the tracking strategy, is compatible with reported biases in direction trajectory and extent for 3D motion targets. Earlier temporal errors caused by MID in both conditions, but specifically in the *immediate feedback* one which is not affected by differential delays, are consistent with perceiving the extent of the motion shorter than it actually was^26^ resulting in earlier responses for larger angles. This effect is thought to be caused by the visual system to process stereo information (e.g., binocular disparity) first and then processing binocular motion^56^. Previously, these biases have been shown in trajectory, extent and speed^14,25–28^ and, to our knowledge, we now show that timing responses can also be affected by MID.

The speed condition also leaves us with some differences that need further discussion. Faster speeds provoke later temporal errors than slower ones as has been reported in previous studies^2,57,58^. However, independently of the tracking strategy, MID increased the difference between speeds. We know that accurate speed estimation does not bode well with 3D motion. Not only is speed in depth underestimated with respect to lateral movement^13,59–61^, but the bias affecting perceived speed in depth might have more effect on larger speeds^14^ under a slow prior interpretation^62^. The difference between speeds is less clear in the *immediate feedback* condition. In this condition, the rate of change of the optical gap between target and interception zone becomes an additional relevant variable^12,63^ that is less affected by possible incomplete mappings from 2D retinal information to a 3D motion and could have been exploited by the participants.

Another possibility is that velocity and position are used differently in these two feedback conditions. Some studies have proposed that response initiation in interceptive timing tasks would be controlled by positional task-relevant variables^2,53,64^ whose internal state could be updated by velocity in a Kalman filter-like way^2^. It could then be that the response is mainly spatially controlled in the *delayed feedback* condition, while velocity would have to be used as well in the *immediate feedback* condition since it becomes a task-relevant variable at the very same moment of the response. This is so because the target speed constraints more clearly the temporal window in which a response has to be executed. Although this interpretation is speculative, there is previous evidence from the TTC literature that is consistent with it. When a coincidence timing task using a single key press was used to estimate the TTC of looming objects participants heavily weighted the rate of expansion, an optical variable which contains spatio-temporal structure^42^. However, in a different study^43^, where the interceptive action did not consist of a key press and movement time could be modulated, participants relied on the visual angle and much less on the rate of expansion.

Another factor that seems to contribute to this incomplete transformation between 2D images and the 3D motion is the tracking strategy. Although we found no significant differences on the final temporal errors due to the tracking strategy participants used, the strategy did affect the response time through its interaction with the angle of approach, that is, when a MID component became more prominent (i.e., larger *β*). People that mainly looked at the end-point showed a trend to respond later, which would be consistent with them perceiving the speed in depth more slowly (Fig. 6A). It is known that MID is perceived more slowly in the periphery^24^ and part of the motion trajectory could have been perceived peripherally when people tracked the end-point. However, this pattern of later responses when the head was oriented towards the shooting area during all the trial (end-point tracking) did not undermine performance and did not significantly affect the temporal error. On the contrary, even increased the proportion of hits in the *immediate feedback* condition significantly because more anticipatory responses when tracking the target reduced the number of hits. Unlike previous studies showing a benefit for tracking^52^, in our study the target trajectory was predictable from exploiting the sensory input and the contribution of extra-retinal signals to speed estimates could have been less relevant^47^. This interpretation based on predictability of target motion^50^ bodes well with studies in which timing performance with 3D trajectories was not affected by gaze strategies^33,48^ or the moment at which the trajectory was seen as long as it was not too late to prepare the response^65^.

Assuming that participants have access to predictable trajectories, previous research has also shown that when participants are required to intercept targets at specific locations, they often rather look at the designated location during the trial or initially look at the target and then direct the gaze towards the indicated location^49,66^. The reasons why directing one’s gaze towards a designated interception location are diverse. One is related to the possible size difference between the target and the interceptive location. If the interceptive location is smaller than the target, it requires a higher spatial precision and thus gaze is directed there^57^ probably to enhance the spatial resolution at that area. In our experiment, both the target and the designated shooting location had the same size, to not favor any of the strategies. By tracking the end-point people increase the spatial resolution by foveating the target around the moment of interest^67^ and reduce the effects that saccades may have in judging how targets move that can give rise to certain perceptual errors^45,46,68–70^ that could lead to errors in interception^6,71^.

In spite of the trajectories being predictable and knowing the shooting location in advance, some participants did track the trajectory with the head leading to a trend of more anticipatory responses in both feedback conditions. It is known that keeping the eyes on the moving target enhances the prediction of visual motion^51^, probably due to additional extra-retinal signals contributing to speed estimation. Anticipatory interception errors have been previously reported in similar interception tasks^5,49,72,73^. Apparently, both when one is allowed to intercept a target anywhere along its path or even when having to do so at an indicated location there is a tendency to hit ahead of the target (errors of ~ 100 ms that become smaller as the precision required to successfully solve the task increases).

## Conclusion

We have reported that MID affects the temporal performance in an interceptive task: the angle of approach of a target with respect to an observer affects the moment of action response in systematic ways. The temporal requirements of the task defined by the type of action effect (delayed or immediate) need to be considered regarding target speed and the amount of MID present in the target trajectory.

## References

[1] Kwon O-S, Tadin D, Knill DC (2015). Unifying account of visual motion and position perception. Proc. Natl. Acad. Sci..

[2] Aguilar-Lleyda D, Tubau E, López-Moliner J (2018). An object-tracking model that combines position and speed explains spatial and temporal responses in a timing task. J. Vis..

[3] Brenner E, Smeets JBJ (2009). Sources of variability in interceptive movements. Exp. Brain Res..

[4] Brouwer A-M, Smeets JBJ, Brenner E (2005). Hitting moving targets: Effects of target speed and dimensions on movement time. Exp. Brain Res..

[5] de la Malla C, López-Moliner J, Brenner E (2012). Seeing the last part of a hitting movement is enough to adapt to a temporal delay. J. Vis..

[6] de la Malla C, Smeets JB, Brenner E (2018). Errors in interception can be predicted from errors in perception. Cortex.

[7] de la Malla C, López-Moliner J (2015). Hitting moving targets with a continuously changing temporal window. Exp. Brain Res..

[8] Cámara C, López-Moliner J, Brenner E, de la Malla C (2020). Looking away from a moving target does not disrupt the way in which the movement toward the target is guided. J. Vis..

[9] Kreyenmeier P, Fooken J, Spering M (2017). Context effects on smooth pursuit and manual interception of a disappearing target. J. Neurophysiol..

[10] Joerges B, López-Moliner J (2019). Earth-gravity congruent motion facilitates ocular control for pursuit of parabolic trajectories. Sci. Rep..

[11] Keil MS, López-Moliner J (2012). Unifying time to contact estimation and collision avoidance across species. PLoS Comput. Biol..

[12] Landwehr K, Hecht H, Both B (2014). Allocentric time-to-contact and the devastating effect of perspective. Vis. Res..

[13] Rushton S, Duke P (2009). Observers cannot accurately estimate the speed of an approaching object in flight. Vis. Res..

[14] Aguado B, López-Moliner J (2019). Perceived speed of motion in depth modulates misjudgements of approaching trajectories consistently with a slow prior. Vis. Res..

[15] Brenner E, van den Berg AV, van Damme WJ (1996). Perceived motion in depth. Vis. Res..

[16] Murdison TS, Leclercq G, Lefèvre P, Blohm G (2019). Misperception of motion in depth originates from an incomplete transformation of retinal signals. J. Vis..

[17] Regan D, Beverley KI (1978). Looming detectors in the human visual pathway. Vis. Res..

[18] López-Moliner J, Brenner E, Smeets JBJ (2004). The role of texture in judging time-to-contact. Perception.

[19] Vincent A, Regan D (1997). Judging the time to collision with a simulated textured object: Effect of mismatching rate of expansion of object size and of texture element size. Percept. Psychophys..

[20] Nefs H, O’Hare L, Harris J (2009). Individual differences reveal two independent motion-in-depth mechanisms. J. Vis..

[21] Nefs HT, Harris JM (2010). What visual information is used for stereoscopic depth displacement discrimination?. Perception.

[22] Harris JM, Nefs HT, Grafton CE (2008). Binocular vision and motion-in-depth. Spatial Vis..

[23] Welchman A, Harris J, Brenner E (2009). Extra-retinal signals support the estimation of 3D motion. Vis. Res..

[24] Brooks K, Mather G (2000). Perceived speed of motion in depth is reduced in the periphery. Vis. Res..

[25] Harris JM, Dean PJA (2003). Accuracy and precision of binocular 3-d motion perception. J. Exp. Psychol. Hum. Percept. Perform..

[26] Lages M (2006). Bayesian models of binocular 3-d motion perception. J. Vis..

[27] Welchman AE, Tuck VL, Harris JM (2004). Human observers are biased in judging the angular approach of a projectile. Vis. Res..

[28] Rokers B, Fulvio JM, Pillow JW, Cooper EA (2018). Systematic misperceptions of 3-d motion explained by Bayesian inference. J. Vis..

[29] La Scaleia B, Zago M, Lacquaniti F (2015). Hand interception of occluded motion in humans: A test of model-based vs. online control. J. Neurophysiol..

[30] Zago M, McIntyre J, Senot P, Lacquaniti F (2008). Internal models and prediction of visual gravitational motion. Vis. Res.

[31] Russo M (2017). Intercepting virtual balls approaching under different gravity conditions: Evidence for spatial prediction. J. Neurophysiol..

[32] de la Malla C, López-Moliner J (2015). Predictive plus online visual information optimizes temporal precision in interception. J. Exp. Psychol. Hum. Percept. Perform..

[33] Aguado B, López-Moliner J (2021). Flexible viewing time when estimating time-to-contact in 3D parabolic trajectories. J. Vis..

[34] Diaz G, Cooper J, Rothkopf C, Hayhoe M (2013). Saccades to future ball location reveal memory-based prediction in a virtual-reality interception task. J. Vis..

[35] Diaz G, Cooper J, Hayhoe M (2013). Memory and prediction in natural gaze control. Philos. Trans. R. Soc. Lond. B.

[36] Wann JP, Rushton S, Mon-Williams M (1995). Natural problems for stereoscopic depth perception in virtual environments. Vis. Res..

[37] Fulvio JM, Ji M, Thompson L, Rosenberg A, Rokers B (2020). Cue-dependent effects of VR experience on motion-in-depth sensitivity. PLoS ONE.

[38] Lee DN (1976). A theory of visual control of braking based on information about time-to-collision. Perception.

[39] Fernandez JM, Farell B (2005). Seeing motion in depth using inter-ocular velocity differences. Vis. Res..

[40] Shioiri S, Saisho H, Yaguchi H (2000). Motion in depth based on inter-ocular velocity differences. Vis. Res..

[41] Tresilian JR (1995). Perceptual and cognitive processes in time-to-contact estimation: Analysis of prediction-motion and relative judgement tasks. Percept. Psychophys..

[42] López-Moliner J, Field DT, Wann JP (2007). Interceptive timing: Prior knowledge matters. J. Vis..

[43] López-Moliner J, Keil M (2012). People favour imperfect catching by assuming a stable world. PLoS ONE.

[44] López-Moliner J, Vullings C, Madelain L, van Beers RJ (2019). Prediction and final temporal errors are used for trial-to-trial motor corrections. Sci. Rep..

[45] Goettker A, Braun DI, Schütz AC, Gegenfurtner KR (2018). Execution of saccadic eye movements affects speed perception. Proc. Natl. Acad. Sci..

[46] Goettker A, Brenner E, Gegenfurtner KR, de la Malla C (2019). Corrective saccades influence velocity judgments and interception. Sci. Rep..

[47] de Xivry J-JO, Coppe S, Blohm G, Lefevre P (2013). Kalman filtering naturally accounts for visually guided and predictive smooth pursuit dynamics. J. Neurosci..

[48] López-Moliner J, Brenner E (2016). Flexible timing of eye movements when catching a ball. J. Vis..

[49] de la Malla C, Smeets JB, Brenner E (2017). Potential systematic interception errors are avoided when tracking the target with one’s eyes. Sci. Rep..

[50] Fooken J, Kreyenmeier P, Spering M (2021). The role of eye movements in manual interception: A mini-review. Vis. Res..

[51] Spering M, Schütz AC, Braun DI, Gegenfurtner KR (2011). Keep your eyes on the ball: Smooth pursuit eye movements enhance prediction of visual motion. J. Neurophysiol..

[52] Fooken J, Spering M (2019). Decoding go/no-go decisions from eye movements. J. Vis..

[53] Brenner E, van Beers RJ, Rotman G, Smeets JBJ (2006). The role of uncertainty in the systematic spatial mislocalization of moving objects. J. Exp. Psychol. Hum. Percept. Perform..

[54] McKee SP, Welch L (1989). Is there a constancy for velocity?. Vis. Res..

[55] Distler HK, Gegenfurtner KR, Van Veen HA, Hawken MJ (2000). Velocity constancy in a virtual reality environment. Perception.

[56] Cumming BG, Parker AJ (1994). Binocular mechanism for detecting motion-in-depth. Vis. Res..

[57] Brenner E, Smeets JBJ (2015). How people achieve their amazing temporal precision in interception. J. Vis..

[58] Brouwer AM, Brenner E, Smeets JB (2000). Hitting moving objects: The dependency of hand velocity on the speed of the target. Exp. Brain Res..

[59] Brenner E, Van Den Berg A, Van Damme W (1997). Perceived motion in depth. Ophthal. Lit..

[60] Brooks KR, Stone LS (2006). Stereomotion suppression and the perception of speed: Accuracy and precision as a function of 3D trajectory. J. Vis..

[61] Welchman AE, Lam JM, Bulthoff HH (2008). Bayesian motion estimation accounts for a surprising bias in 3D vision. Proc. Natl. Acad. Sci. USA.

[62] Stocker AA, Simoncelli EP (2006). Noise characteristics and prior expectations in human visual speed perception. Nat. Neurosci..

[63] Bootsma R, Oudejans R (1993). Visual information about time-to-collision between two objects. J. Exp. Psychol. Hum. Percept. Perform..

[64] Wann JP (1996). Anticipating arrival: Is the tau-margin a specious theory?. J. Exp. Psychol. Hum. Percept. Perform..

[65] López-Moliner J, Brenner E, Louw S, Smeets JBJ (2010). Catching a gently thrown ball. Exp. Brain Res..

[66] de la Malla C, Rushton SK, Clark K, Smeets JB, Brenner E (2019). The predictability of a target’s motion influences gaze, head, and hand movements when trying to intercept it. J. Neurophysiol..

[67] Schütz AC, Braun DI, Gegenfurtner KR (2009). Object recognition during foveating eye movements. Vis. Res..

[68] Schlag J, Schlag-Rey M (2002). Through the eye, slowly: Delays and localization errors in the visual system. Nat. Rev. Neurosci..

[69] Matziridi M, Brenner E, Smeets JB (2015). The role of temporal information in perisaccadic mislocalization. PLoS ONE.

[70] Morrone MC, Burr DC, Vaina LM (1995). Two stages of visual processing for radial and circular motion. Nature.

[71] de la Malla C, Brenner E, de Haan EH, Smeets JB (2019). A visual illusion that influences perception and action through the dorsal pathway. Commun. Biol..

[72] de la Malla C, López-Moliner J, Brenner E (2014). Dealing with delays does not transfer across sensorimotor tasks. J. Vis..

[73] Brenner E, Cañal-Bruland R, van Beers RJ (2013). How the required precision influences the way we intercept a moving object. Exp. Brain Res..

